# Advances in the support of respiratory failure: putting all the evidence together

**DOI:** 10.1186/cc14722

**Published:** 2015-12-18

**Authors:** John J Marini

**Affiliations:** 1 University of Minnesota, Minneapolis/St. Paul, MN, USA

## Abstract

Considerable progress has been made recently in the understanding of how best to accomplish safe and effective ventilation of patients with acute lung injury. Mechanical and nonmechanical factors contribute to causation of ventilator-associated lung injury. Intervention timing helps determine the therapeutic efficacy and outcome, and the stage and severity of the disease process may determine the patient's vulnerability as well as an intervention's value. Reducing oxygen consumption and ventilatory demands are key to a successful strategy for respiratory support of acute respiratory distress syndrome. Results from major clinical trials can be understood against the background of the complex physiology of ventilator-induced lung injury.

## Introduction

In recent years physicians have learned to care better for their patients with acute respiratory failure. For no condition has this improvement become more evident than in the treatment of acute respiratory distress syndrome (ARDS), a signature problem of intensive care. Over time, we have learned important lessons. We know from research and experience that despite well-intentioned attempts at revision, our clinical definition for ARDS still needs work. We are now also aware that many factors--mechanical and nonmechanical--contribute to ventilator-induced lung injury (VILI). Timing has proven key to the efficacy of several important ventilation-related therapeutic interventions. Moreover, the stage and severity of disease determine an intervention's potential value or harm. A collective implication of the growing evidence base related to respiratory failure is that reducing oxygen consumption and ventilator demands is a rational point of focus and high priority when attempting to optimize respiratory support for the individual patient.

## Definition of ARDS

When ARDS was first defined by Ashbaugh, Bigelow, and Petty in 1967 [1], they envisioned a disease of adults caused by diverse insults but with pathophysiology innately similar to the infant respiratory distress syndrome (IRDS)--at least in the sense that surfactant deficiency was key to injury generation and/or perpetuation. These pioneering authors recognized that although many conditions resulted in acute lung injury, all evolved from initial permeability edema through an intermediate stage of cellular proliferation to its resolution by healing or fibrosis. The clinical presentation of that disorder was based on the awareness of noncardiogenic, high-permeability edema associated with stiff lungs, abrupt onset, diffuse infiltrates, and refractory hypoxemia that often responded to positive end-expiratory pressure (PEEP). Indeed, Tom Petty [2] stated in a later paper that a favorable blood gas response to PEEP should be part of the definition of this syndrome. The concept that positive pressure ventilation could itself cause injury that mimics ARDS had not yet gained traction.

From that early time to this we have continued to espouse the concept that different insults lead to a shared pathophysiology which defines a clinical syndrome that justifies a unified clinical approach. It is interesting, however, that the original 12-patient cohort Petty and colleagues described might not have initially had ARDS as we currently understand it to be. Five of the 12 original patients were managed with nasal oxygen, room air, or low-flow oxygen masks [1]. It is highly likely that even a modest amount of PEEP would have eliminated those five patients from classification by current criteria, which are based primarily on disordered gas exchange. Further examination of tabulated data from that original cohort demonstrates that several patients were clearly fluid overloaded. It is unsettling to remember that once intubated, the ventilation standard of the time prescribed very high tidal volumes and minimal PEEP--just the formula for potentiating VILI [3,4]. Like those earliest groundbreakers, present-day practitioners continue to concentrate on gas exchange as the primary criterion for identification of ARDS. Consequently, in our clinical research we continue to mix together patients who do not necessarily share key underlying pathologic characteristics of lung injury.

**Figure 1:**
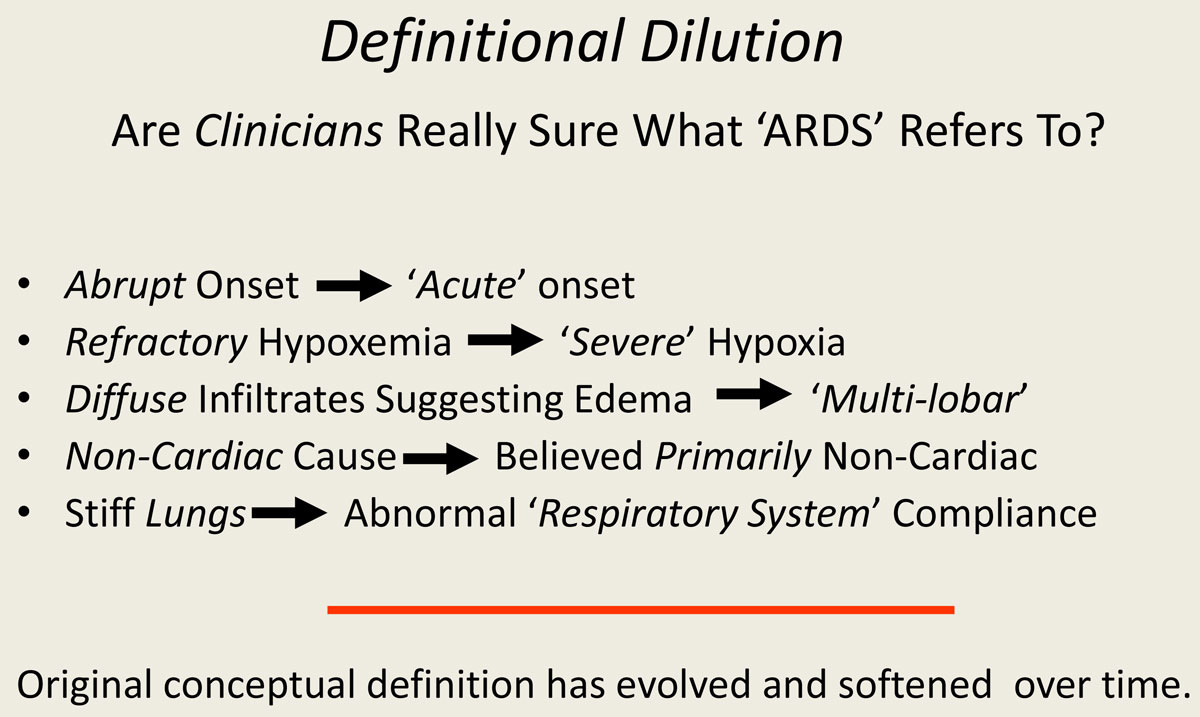
**Definition of ARDS: changes over time**.

Debate regarding the concept that a unifying entity of ARDS even exists was conducted throughout the 1970s and into the 1980s [5,6]. During that latter decade, suspicion grew that the therapy we applied for life support in an attempt to improve oxygenation could itself cause damage to the lung or retard its healing (VILI) [3]. Moreover, those years saw the rise of evidence-based medicine (EBM) and of the perceived need for randomized clinical trials (RCTs) to provide a convincing and reliable basis for making care decisions [7]. Motivated by those developments, the American-European Consensus Committee (AECC) formulated a definition of ARDS in 1994 [8], and this standard served as the qualifying definition for numerous trials of respiratory support, most of which proved inconclusive [9]. That AECC definition did not include specific criteria relating to respiratory mechanics or stipulate precise radiographic criteria. The gradually recognized need for improved discriminating criteria resulted in the Berlin-modified version of the AECC definition, published in 2012 [10]. Although clearly an improvement over the AECC definition, the need to interface with data from prior clinical trials resulted in shared features that continue to be overly broad and inexact (Figure 1). No mechanics criteria were included, no standard ventilatory settings were specified, radiographic criteria remain imprecise, no requirement was made for confirming the durability of the syndrome over time, and there was no accounting for nonpulmonary contributors to hypoxemia (e.g., excessive oxygen extraction) that might be unrelated to inflammatory conditions within the lung itself. Functional tests are lacking in the current Berlin definition. At the bedside, one might wonder whether clinicians are really sure what the Berlin-defined term "ARDS" now refers to. When comparing Petty's original definition with the current one, we note that "abrupt" onset has become "acute" onset whose putative cause might have originated as long as 1 week earlier. Some terminology of the initial 1967 definition has been relaxed in other ways: "refractory" hypoxemia has become "severe" hypoxemia, "diffuse" infiltrates have become "bilateral multi-lobar" infiltrates, and "stiff lungs" are now "reduced respiratory system compliance"--a term that includes the chest wall.

With guidance from such imprecise definitions, most trials undertaken for therapy of ARDS have failed to demonstrate new interventions, pathways, and strategies that consistently improve care [9]. Yet, despite our imprecise definitions, we have made good progress in the outcomes emerging from our bedside management. The overall mortality attributable to ARDS has fallen impressively in the past decades [11]. Certainly, some of this declining mortality has resulted from reducing the tidal volume and the associated driving pressures (the ratio of tidal volume to compliance) that produce VILI [12]. But declining mortality is not just the result of less VILI; mortality has continued to fall impressively, even as tidal volumes are kept low to mid-range in each arm of later therapeutic trials [11]. One likely contributor to this improvement was growing awareness of the damaging potential of high airway pressure and the value of lung recruitment. Such information gradually seeped from the laboratory into clinical practice, well before the confirmatory RCT. Advances are the result not only of improvements in the process and delivery of care to our patients who require respiratory support, but also in the avoidance of volume overload [13], unnecessary transfusions [14], and ventilator-associated pneumonia [15], among other potentially iatrogenic interventions.

Perhaps because we have used imprecise definitions, we are left at the bedside with long simmering questions related to the mechanical ventilation and ventilatory support of patients with lung injury. For the individual patient, these questions include: which tidal volume should we use? Which PEEP should we use? Should we employ recruiting maneuvers? Should prone positioning be routine? Is high-frequency ventilation lung protective or damaging? Should we encourage spontaneous breathing in the early stage of illness, or take control of the breathing pattern? Should we use corticosteroids in our patients with inflammatory lung disease? Should we embrace extrapulmonary gas exchange technology, and if so, in whom? The list of unsettled questions can be made longer.

## Lessons from the laboratory

Management principles derived from laboratory results appear much clearer. Experimental evidence provides consistent laboratory observations regarding what is needed to avoid inflicting VILI. Adverse patterns of ventilation injure both airways and alveoli, causing damage that prevails in anatomically dependent zones. Laboratory experiments have shown that adverse patterns of ventilation apply large tidal volumes with high driving pressures, low levels of PEEP, high inspiratory flows, and elevated minute ventilations. Nonmechanical background factors are also important in the process of VILI generation and, for any given ventilation pattern that applies potentially damaging stress, may determine whether or not VILI is expressed. These background factors, which may synergize with each other, include the preinjury and inflammatory state, the temperature at which the experiment is conducted [16], the amplitudes of vascular pressures and flows [17,18], body position [19], PaCO_2_/pH [20], and FiO_2 _[21]. The mechanisms of airspace injury that mediate adverse patterns of mechanical ventilation include stretching of open lung units, amplified tangential (shearing) forces at the interface between open and closed lung units, and the recurring small airway trauma of tidal breathing.

The interplay of mechanical stressors that determine whether or not tissues are injured by the ventilation pattern involves an interaction between driving pressure, defined as the difference between plateau pressure and PEEP, and the number of lung units at higher risk for stress amplification; that is, those at the junctions of open and closed lung tissue. Although dynamic characteristics are less well studied as provocative influences, the rate at which the lungs are expanded, determined by the amplitude and pattern of airflow delivery, may be an important determinant of the ventilator-associated damage that results from the tidal excursion. The focus of investigative attention regarding VILI has been on the individual tidal cycle--as defined by PEEP and tidal volume. However, it stands to reason that the number of damaging cycles delivered per unit time (closely correlated with minute ventilation, independently of mode) would accentuate the injury inflicted by the individual tidal cycle [22]. PEEP tends to reduce the number of lung units placed at high risk by critical junctional interfaces between expanding and reluctantly expanding tissues. For the same tidal volume, PEEP also elevates the mean airway pressure, and with it the average tissue stress. In the absence of compensatory recruitment or reduction in tidal volume, PEEP therefore also tends to increase right ventricular afterload. Without a simultaneous reduction in driving pressure, raising PEEP will place the lung at higher risk for stretch-related injury. It should be noted that the tidal volume itself may not injure the ventilated lung, but rather, the causative variable relates to the ratio of the tidal volume to the capacity of the lung to accept it [23]. The transalveolar pressure and the swings of transalveolar pressure (transalveolar driving pressure) determine the damaging energy forces imparted to delicate tissue. In this discussion, it is important to note that the pressures traditionally used in clinical practice to judge VILI hazard--all based on airway pressure alone--might not be sufficient to gauge risk for injury when the chest wall is abnormally stiff or the patient breathes actively. The same plateau pressure, PEEP, and driving pressure measured at the airway opening may be associated with dramatically different transalveolar pressures and swings of transalveolar pressure when the surrounding pleural pressure is taken into account. Conditions that often modify the impact of a given plateau or driving pressure are exemplified by morbid obesity, intra-abdominal hypertension, and vigorous spontaneous breathing.

While the use of the esophageal balloon catheter as a sensor of intrapleural pressure is helpful and offers a definite clinical advantage over airway pressures alone for some purposes (e.g., determining lung compliance and stress), esophageal pressure (Pes) measurements are not entirely representative of all transalveolar environments encountered locally (site to site) throughout the lung [24]. For example, negative values of transpulmonary pressure, calculated as alveolar pressure minus esophageal pressure, suggest that lung units are collapsed at the same horizontal level as the catheter's balloon--but not throughout the aerated portions of the lung, where positive values of transpulmonary pressure would be recorded.

There are several theoretical advantages of using an esophageal balloon catheter in our most seriously lung-injured patients. The balloon catheter is a relatively noninvasive probe that helps monitor transpulmonary static and dynamic pressures under both active and passive conditions. Furthermore, the balloon catheter samples pleural pressure in an important "interface" (mid-lung) zone that is highly predisposed to VILI. Lung units at the interface between open and closed tissue undergo regional stress focusing and force amplification that may incite inflammation or overtly tear microvessels and alveolar epithelium. The damaging energy delivered to lung tissue per unit time (power) is locally higher there.

Esophageal pressures must be carefully recorded and interpreted. Calculations based on Pes are not always representative of true risk and Pes is susceptible to artifacts from its own local environment and from the weight of the structures that lie above it. Transpulmonary pressure may increase either if the lung volume changes or if compliance declines. In part for this reason, transpulmonary pressure does not closely track absolute lung volume changes that result from rising intra-abdominal pressure at end expiration (when the circuit is open and the lung is free to lose or gain units). The Pes-determined estimate of pleural pressure may not reflect those lung units that are most stretched or those already collapsed, as the Pes-based trans-pulmonary pressure (Ptp) is only regionally valid and tracks only the aerated compartment of the lung [25]. These weaknesses were made strikingly evident by experiments conducted with changing position and mechanically asymmetrical lung disease [26,27].

Given the advantages and disadvantages, the key uses for Pes determinations are to: set an effective PEEP level that maintains the lung less de-recruited [28]; to better approximate the relevant driving pressure to which the lung is exposed; and to guide the clinician in selecting pressures which will avoid excessive transpulmonary pressure during both passive and spontaneous breathing.

## Cofactors of VILI

When the mechanical stresses of the tidal cycle are high, experiments have shown that an increase in precapillary vascular pressure or a reduction in postcapillary vascular pressure each accentuate VILI [17]. These observations imply that the gradient of transalveolar vascular pressure may be instrumental in inflicting damage when airway stresses are high. Other experiments indicate that an increase of body temperature dramatically increases the tendency for and severity of VILI to be expressed. Experimentally, the metabolic/oxidative/pH environment of the lung tissue at the onset of major mechanical stress is influential. The results of such controlled laboratory experiments suggest that lowering ventilatory and cardiovascular demands--thereby reducing both minute ventilation and the vascular pressure gradient across the lung--are important therapeutic levers when attempting to avoid VILI. Because creation of dead space increases the ventilation needed for CO_2 _elimination, clinical data that demonstrate a strong correlation between alveolar dead space and mortality risk would seem consistent with the admonition to reduce the vigor of ventilation support by reducing demands [29]. In recent years, the deployment of bedside extrapulmonary gas exchanging technology to remove CO_2 _and oxygenate the venous blood has offered long-awaited and much-needed assistance in accomplishing this objective.

Perhaps no issue has been more troublesome to the physician attempting to select and regulate the ventilator's prescription than selecting the appropriate level of PEEP. Insufficient PEEP allows unnecessary collapse of recruitable tissue, whereas excessive PEEP promotes tissue stretch and dead space generation while elevating the mean airway pressure and right ventricular afterload. Unless the tidal volume is simultaneously reduced, increasing PEEP also elevates the plateau pressure. When the overstretching of open lung tissue outweighs the benefit from recruitment, PEEP redirects pulmonary blood flow and accentuates mechanical heterogeneity within the acutely injured lung. Although either respiratory system compliance or oxygenation can be used as the criterion target for PEEP selection with good physiological justification, it appears as if oxygenation response best parallels stabilized recruitment of collapsed lung units, which is the primary objective for PEEP employment [30].

Recruitment of lung tissue can be attained without increasing PEEP by prone positioning. Because modifications of the chest wall that result from prone positioning help even the distribution of transpulmonary pressures, several of the important disadvantages of PEEP just enumerated can be avoided. Prone positioning tends to recruit selectively in dorsal regions that are gravitationally dependent when supine. Airway secretion drainage is also promoted.

**Figure 2:**
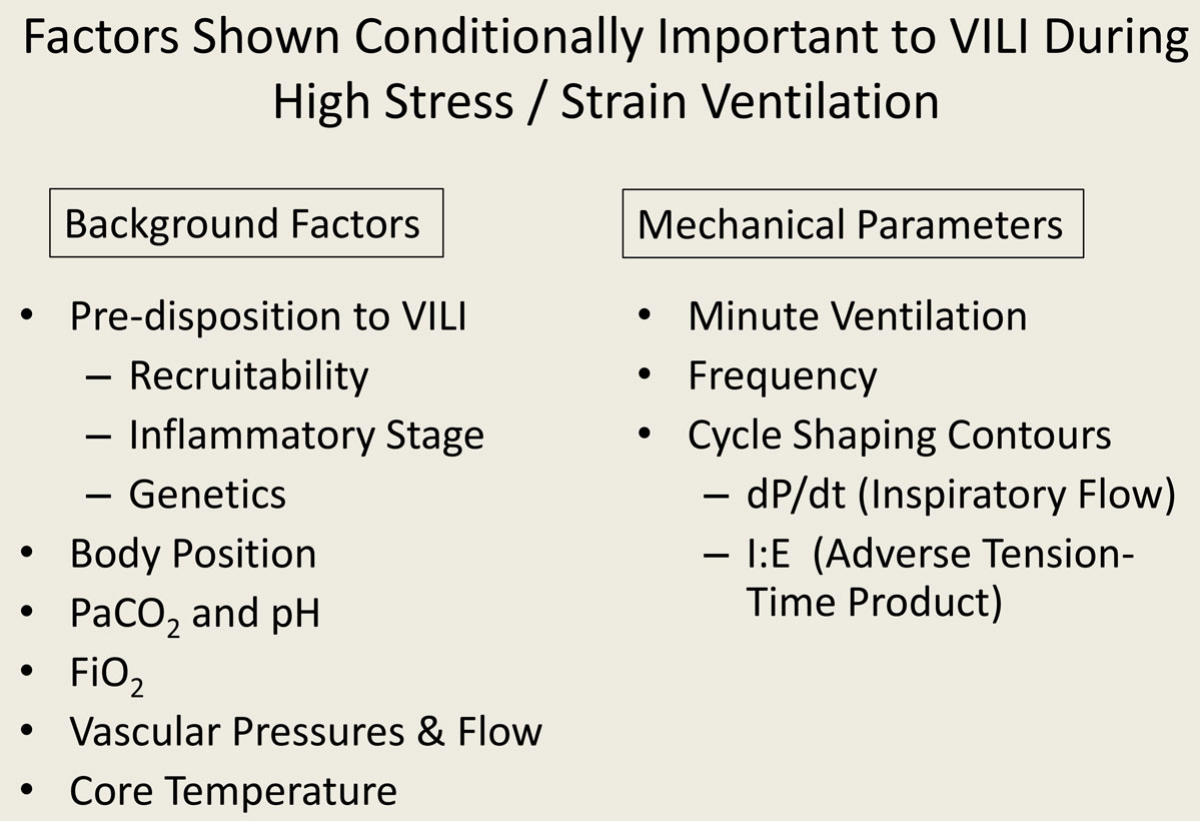
**Conditional co-factors of VILI**.

A key lesson learned from investigations conducted over the past three decades is that excessive stress and strain are essential prerequisites for inflicting lung damage, but are only the preconditions to VILI (Figure 2). As already noted, both the vascular compartment and the airspace compartment are subject to damaging forces generated during lung expansion. Reducing oxygen demand and ventilation requirement are essential components of a comprehensive "lung protective" strategy. Reducing the ventilation requirement simultaneously allows reduction of driving pressures or ventilating frequencies, or both. These benefits underpin the rationale for permissive hypercapnia. Because cardiac output also declines in response to lower oxygenation demands, the pulmonary microvascular blood flow gradient is lessened, reducing VILI risk. In recent years the bedside deployment of safe and effective extracorporeal gas exchange has provided the great advantage of accomplishing CO_2 _removal upstream from the lungs themselves, offloading much of the dynamic burdens of ventilation.

## Clinical trials of respiratory support in ARDS

**Figure 3:**
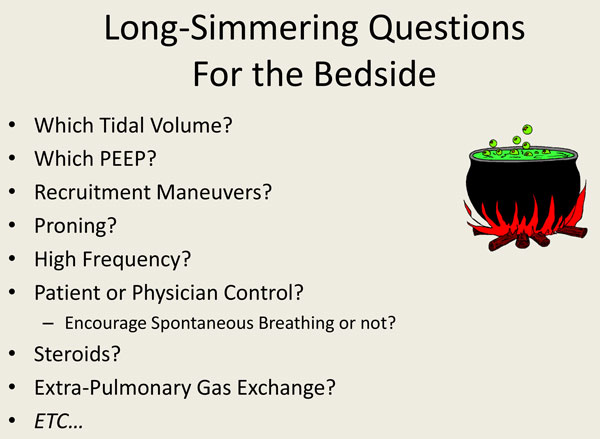
**Unresolved bedside questions regarding ventilator support**.

For more than two decades, clinicians have used standardized definitions of ARDS in RCTs of interventions aimed at learning what physicians can do to stop VILI [8,10]. Unfortunately, the majority of these RCTs have proven inconclusive and/or discouraging (Figure 3). In fact, only three large trials have convincingly shown definitive findings [9]. Meta-analyses have demonstrated that the likely explanation for the disappointing results of many of the other trials may be explained by the composition of the study sample or by the timing of the intervention. Using the exceptionally consistent laboratory base regarding mechanical principles of lung protection, many of the key results stemming from RCTs can be explained. Among the most influential of the RCTs was the ARMA trial of the National Institutes of Health-sponsored Acute Respiratory Distress Syndrome Network investigators, which showed that smaller tidal volumes were safer to employ than larger ones [31]. This result is hardly unexpected; in a given individual, reducing tidal volume results in a smaller and often less dangerous driving pressure (a key determinant of VILI) [12,32]. The latter parameter, which can be measured with relative ease at the bedside as the difference of plateau pressure and total PEEP during passive inflation, scales tidal volume to respiratory system compliance. Embedded within the Positive End Expiratory Pressure Setting in Adults with Acute Lung Injury and Acute Respiratory Distress Syndrome (EXPRESS) [33] and Lung Open Ventilation Study (LOVS) [34] trials of high versus low PEEP are data which indicate lower mortality results using higher PEEP when it is associated with an unchanging plateau pressure and reduced driving pressure, especially in patients with disease of higher severity treated earlier in their disease course. Conversely, higher mortality tends to result from using higher PEEP in patients with lower severity and less recruitability. This adverse outcome presumably reflects more tissue stress and higher right ventricular afterload in response to unnecessary lung distention. Two trials of high-frequency oscillatory ventilation (HFO) have produced unexpected results that discourage its routine use [35,36]. One plausible explanation, among others, is that the higher mean airway pressure generated by oscillatory ventilation presented increased and possibly cor pulmonale-inducing afterload to the right ventricle, nullifying any potential benefit of modest recruitment. The Oscillation for Acute Respiratory Distress Treated Early (OSCILLATE) trial that convincingly showed harm from HFO imposed higher mean airway pressures than did the OSCAR (High Frequency Oscillation in Acute Respiratory Distress Syndrome) trial, which simply showed equivalence between HFO and standard lung protective ventilation in a less severely affected population supported at more modest mean airway pressure levels [35,36].

A recent bright spot in trials of ventilation strategy was the demonstration that prone positioning could help very severely ill patients early in the course of illness when recruitability was presumably higher [37]. As the Prone Positioning in Severe Acute Respiratory Distress Syndrome (PROSEVA) study by Guérin and colleagues [38] nicely demonstrated, prone positioning sustained for 12 hours or more early in the course may help those who are severely affected and who have high PEEP requirements in the supine orientation. Glimmerings of the same positive effect for the early/severe cohort were seen in the first large Italian trial testing this same question [39], which also suggested the caution that some patients who are neither severely ill nor recruitable may not receive benefit but actually may be harmed by the proning intervention.

Another positive trial that relates to mechanical ventilation, the Neromuscular Blockers in Early Acute Respiratory Distress Syndrome (ACURASYS) study found benefit from the use of muscle relaxants early in the disease course [40]. This finding ties nicely into the concept that transalveolar pressure must be minimized and that spontaneous ventilation has high potential to be damaging early in the disease course. It must be remembered that when patients have high demands, vigorous breathing violates the objectives of lung protection for multiple reasons. The work of breathing moderately increases cardiac output and pulmonary vascular flow. More importantly, not only are transpulmonary inspiratory stresses high, but during exhalation the muscular effort often compresses the thorax below the PEEP-appropriate functional residual capacity (FRC). This not only encourages de-recruitment but also increases effective inspiratory transpulmonary forces that drive inspiration. When end-expiratory relaxation occurs, an inflationary bias is applied to combine with inspiratory pressure. Rapidly accelerating flow and inspiratory flow (and the rates of change of pressure they generate (dP/dT)) cause major stress on dependent lung zones most predisposed to VILI. Here again, the timing of an intervention may determine its value. Spontaneous breathing efforts should be encouraged if the disease severity and demands are modest or the patient is later in the support period. Spontaneous breathing efforts are inadvisable early on in the treatment course of severe illness, or when high ventilation demands or specialized treatments such as prone positioning or cooling are needed. It is also worth considering that we may not always trust the "wisdom of the body" [41]; the natural response to severe illness may be to accentuate a catastrophic problem rather than resolve it. Taking control in this critical early phase may interrupt a self-destructive downward spiral. The apparent disconnect regarding the timing of neuromuscular blocking agent (NMBA) administration (first 48 hours) and the mortality benefit (separation occurring only after 2 weeks) observed in the ACURASYS trial seem consistent with this possibility [40].

## Summary

**Figure 4:**
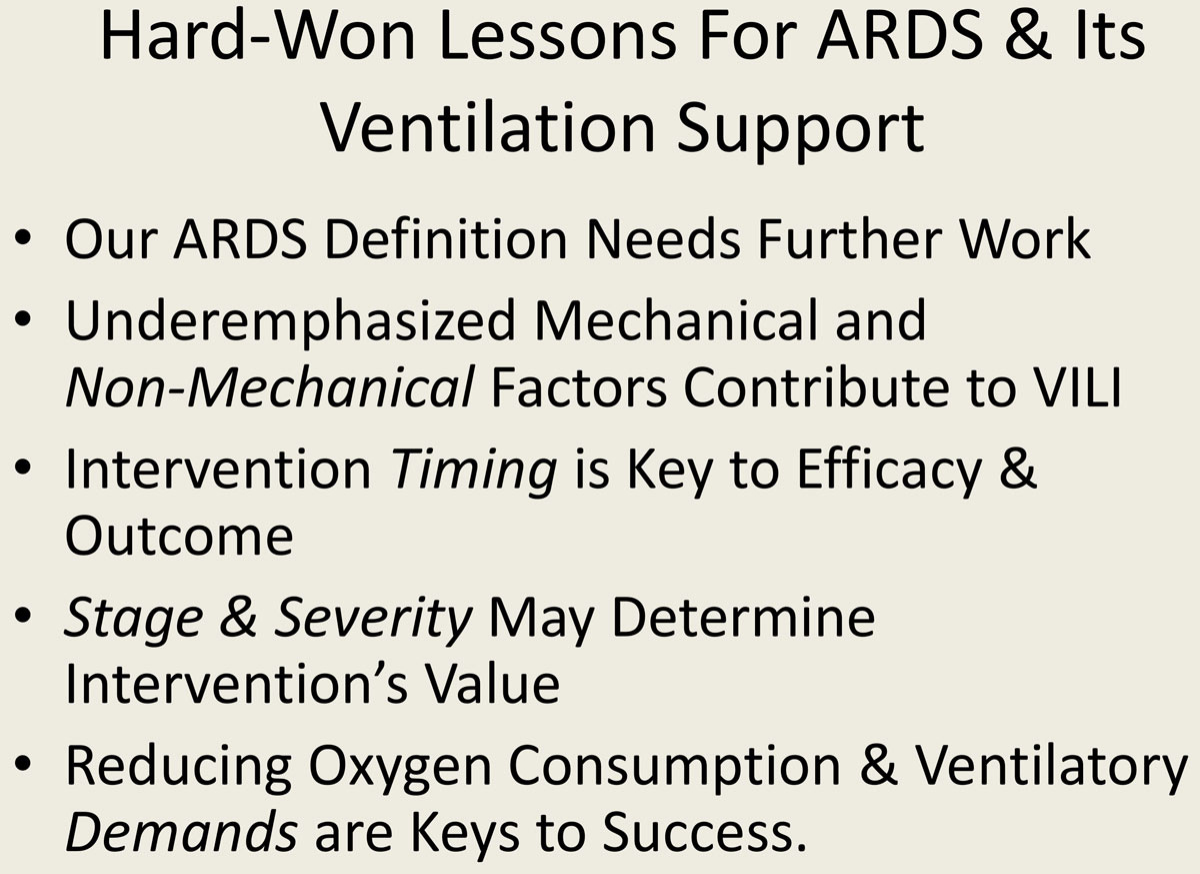
**Important principles for the ventilatory support of ARDS**.

As noted at the beginning of this discussion, we have learned some hard won lessons (Figure 4). Among these, we know that our ARDS definition needs considerable work in order to personalize ventilatory care for patients with acute lung injury and distress). Mechanical and non-mechanical factors contribute to causation of ventilator-associated lung injury. Intervention timing is key to the efficacy and outcome, and the stage and severity may determine an intervention's value. Reducing oxygen consumption and ventilatory demands are keys to a successful strategy for respiratory support of ARDS. Results from major clinical trials can be understood against the background of the complex physiology of VILI. Unquestionably, our laboratory investigations have been informative, whereas our clinical progress in managing ARDS has been slow, sometimes painful, but nonetheless real.

## Abbreviations

AECC, American-European Consensus Committee; ARDS, Acute respiratory distress syndrome; EBM, Evidence-based medicine; HFO, High-frequency oscillatory ventilation; IRDS, Infant respiratory distress syndrome; PEEP, Positive end-expiratory pressure; RCT, Randomized clinical trial; VILI, Ventilator-induced lung injury.

## Competing interests

The author declares that he has no competing interests.

## Notes

This article has been published as part of *Critical Care *Volume 19 Supplement 3, 2015: Future of Critical Care Medicine (FCCM) 2014. The full contents of the supplement are available online at http://www.ccforum.com/supplements/19/S3. Publication of this supplement was supported by Fresenius Kabi.
